# Investigating the impact of peatland degradation: A lipid biomarker analysis

**DOI:** 10.1016/j.isci.2025.112604

**Published:** 2025-05-07

**Authors:** Nasreen Jeelani, Katharina Fischer, Carrie L. Thomas, Klaus-Holger Knorr, Mariusz Lamentowicz, Mariusz Gałka, Stephan Glatzel

**Affiliations:** 1 University of Vienna, Faculty of Earth Sciences, Geography and Astronomy, Department of Geography and Regional Research, Working Group Geoecology, Josef-Holaubek-Platz 2, Vienna 1090, Austria; 2 Radboud University, Department of Ecology, Nijmegen, the Netherlands; 3 Institute for Landscape Ecology, Ecohydrology and Biogeochemistry, University of Münster, 48149 Münster, Germany; 4 Climate Change Ecology Research Unit, Adam Mickiewicz University, Poznań, Bogumiła Krygowskiego 10, Poznań 61-680, Poland; 5 University of Lodz, Faculty of Biology and Environmental Protection, Department of Biogeography, Paleoecology and Nature Conservation, Łódź, Poland

**Keywords:** Environmental science, Environmental chemistry

## Abstract

Lipids, such as aliphatics, phenolics, and carboxylic acids, are key biomarkers for analyzing peat composition, decomposition, and microbial activity. This study provides a comparative analysis of lipid biomarkers from natural, extracted, degraded, and restored bog sites in Germany, Poland, and Austria. We investigate samples from various depths, assessing how effectively these biomarkers reflect environmental conditions and restoration success. The aliphatic hydrocarbon fractions showed the site specific pattern in *n*-alkanes (C_15_ to C_33_), with variations in carbon preference index (CPI), average chain length (ACL), and proxy of aquatic macrophytes input (P_aq_) indicating shifts in vegetation and moisture. The presence of triterpenoids, sterols, hopanoids, and archaeol provides insights into the vegetation composition and microbial processes. Our finding highlights the complex interaction of chemical and biological factors in peatlands. Restoration sites exhibit elevated biomarker concentrations similar to natural conditions, whereas degraded sites show lower concentrations, suggests additional restoration efforts are needed to achieve natural condition.

## Highlights

• Peat samples from natural, extracted, degraded, and restored bogs were examined
• Lipid biomarkers reveal changes in vegetation and environmental conditions at peat sites
• Restoration efforts have successfully promoted vegetation recovery at certain sites
• Degraded sites still need further restoration to achieve natural conditions

## Graphical abstract

**Figure:**
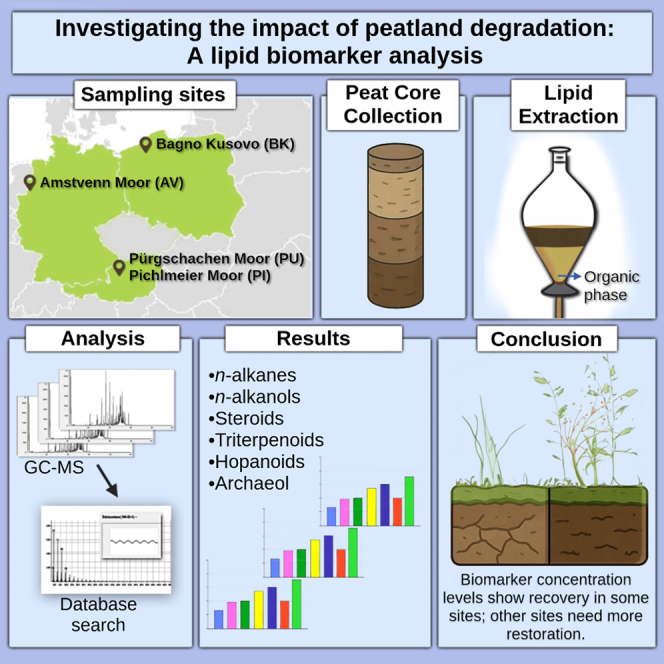

## Introduction

Peatlands, which cover 3%–5% of the earth’s land surface, store around 30% of the global soil carbon. Collectively, they are the largest and most spatially efficient terrestrial carbon sinks.^1^^,^^2^ Peatlands are vital for enhancing ecosystem resilience and supporting local livelihoods.^3^^,^^4^^,^^5^ During the 20th century, peatlands were subject to anthropogenic alteration on a large scale, and many were drained for agricultural and industrial reasons.^6^ According to data from the Greifswald Mire Center,^7^ a total of 527,783 km^2^ of peatland has been drained worldwide, with the majority of this drainage occurring in Europe. In recent years, there has been increasing interest and awareness toward revitalizing degraded peatlands to restore biodiversity and hydrology and mitigate carbon dioxide (CO_2_) emissions to the atmosphere.^8^^,^^9^

Peatlands are characterized by the accumulation of organic matter over time as peat, derived from dead and partially decomposed plants.^10^^,^^11^ However, microbial biomass also plays an important role in peat composition, contributing to organic matter through microbial activity and residues.^12^ The composition of peat can thus provide information about variations in former plant and microbial communities and provide site-specific records of *in situ* environmental histories.^13^ The unique hydrology and chemistry particular to ombrotrophic peatlands define distinct plant communities, making them responsive and vulnerable to climate change as abiotic conditions in peatlands are affected by warming. Investigating past vegetation dynamics and peat accumulation can thus not only help in understanding peatland past development but is also critical for understanding future responses to warming and potential impacts on ecological functions.^14^

The complex balance between plant decomposition and peat accumulation is closely related to the water table level (WTL) within the profile. Decomposition rates peak in the acrotelm, where aerobic microbial communities facilitate rapid degradation of complex organic matter.^12^ As conditions shift from oxic to anoxic in the catotelm, decomposition rates gradually decrease with increasing depth below WTLs.^15^^,^^16^^,^^17^^,^^18^ A range of proxies can be extracted from peat to reconstruct palaeo-environmental and biogeochemical conditions. Classical proxies, such as plant macrofossils and pollen directly identify vegetation shifts, which in turn provide insights into historical variations in temperature and precipitation.^19^^,^^20^ Testate amebae, protozoa that prefer aquatic to moist habitats, serve as another type of proxy used in peat analysis to reconstruct historical moisture conditions and climate variations.^21^ Although these proxies have provided significant insights into past climatic conditions, they are sometimes difficult to apply to ancient peats that are highly humified.^22^^,^^23^ Among further proxies, biomarkers have thus gained significant attention.^24^ These stable and long-lived compounds have proven particularly valuable to trace the geological and past environmental changes of peatland ecosystems.^25^^,^^26^^,^^27^

Lipids, including aliphatics, phenolics, and carboxylic acids, are widely recognized as reliable biomarkers for analyzing peat qualitative composition and origin, degree of decomposition, and microbial contributions.^22^^,^^28^^,^^29^ Aliphatic hydrocarbons including *n*-alkanes, are particularly resistant to diagenesis,^30^ and this, along with their source specificity, makes them reliable biomarkers for tracing the origin of organic matter.^31^^,^^32^ For instance, algae and photosynthetic bacteria are the primary source of short-chain *n*-alkanes (C_15_–C_21_)^33^^,^^34^ while submerged and emergent aquatic plants predominantly produce medium-chain *n*-alkanes (C_21_, C_23_, and C_25_).^35^^,^^36^ Long-chain *n*-alkanes (C_27_–C_33_) are indicative of terrestrial plants.^37^^,^^38^ The specific chain lengths of *n*-alkanes therefore serve as indicators of the source of organic matter.^39^ In addition, triterpenes such as sterols (derived from eukaryotes) and hopanoids (derived from prokaryotes) are emerging as valuable tools to identify plants, algae, and bacterial contributions, reconstruct past environmental conditions, and, by this, guide restoration efforts, providing insights into the original state and ecological dynamics of peatlands.^22^^,^^32^^,^^40^^,^^41^

Despite the usefulness of these novel indices and indicators in providing insight into past environmental conditions, there remains a significant gap in understanding the organic geochemistry of peat across different site history conditions. Our comprehensive study addresses this gap by including samples from near-natural, degraded, and restored ombrotrophic bog sites across three European countries: Amtsvenn (Germany), Bagno Kusowo (Poland), and Pürgschachen Moor and Pichlmaier Moor (Austria). We focus on near-natural areas and those restored after prior peat cutting, particularly in Bagno Kusowo and Pürgschachen Moor. In Amtsvenn, we examine degraded sites under drained conditions, both with and without prior peat cutting. At these specific sites, detailed information on the organic geochemistry of the peat is currently insufficient. Consequently, this study has been undertaken to determine the composition and origin of lipid biomarkers in the peat bogs within these diverse ecosystems at different depths. This study aims to test these biomarkers in a range of sites to elucidate their specific suitability for inferring restoration success in different systems, which is essential for improving peatland management and restoration strategies, particularly in the context of ongoing environmental changes.

### Methodology and materials

#### Study sites

In this study, we investigate three different locations: Amtsvenn, Germany, Pichlmaier Moor and Pürgschachen Moor, Austria and Bagno Kusowo, Poland. These sites were selected chosen because they represent a range of degraded and restored conditions. At each location, we chose two study sites (Table 1) and sampled peat from 10, 30, and 80 cm depth to analyze the biomarker signals. The depths were chosen to include distinct peat layers with differing redox conditions: the 10 cm depth corresponds to the acrotelm, which is primarily in oxic conditions; the 80 cm depth represents the catotelm, which remains permanently waterlogged and anoxic, even at the drained sites under study; and the 30 cm depth represents the zone between the acrotelm and catotelm, where water table fluctuations can lead to change between oxic and anoxic conditions.

**Table 1:** Geographical location of study sites and their environmental status

Site name	Status	pH	WTL (cm)	Terms	Coordinates
Amtsvenn (AV)	Drained/degraded	4.3	−43	AV-D	52°10′31″*N* – 06°57′22″E
Amtsvenn (AV)	Extracted/degraded	4.3	−50	AV-ED	52°10′31″*N* – 06°57′20″E
Pürgschachen (PU)	Near natural/well preserved	4.12	−17	PU-N	47°34′50″*N* – 14°20′50″E
Pichlmeier (PI)	Extracted and restored	4.23	−7	PI-ER	47°34′49″*N* – 14°24′55″E
Bagno Kusowo (BK)	Near-natural/well preserved	3.75	−11	BK-N	53°48′54″*N* – 16°35′20″E
Bagno Kusowo (BK)	Extracted and restored	3.75	−18	BK-ER	53°48′45″*N* – 16°35′12″E

##### Amtsvenn-Hündfelder Moor

According to Sevink et al.,^42^ the bog “Amtsvenn-Hündfelder Moor,” termed “Amtsvenn” here and abbreviated “AV”, located in North-Rhine Westphalia, Germany, near the Dutch border, reached its greatest extent in the 17th century. Significant peat extraction and agricultural reclamation began in the early 19th century, particularly for buckwheat cultivation, and the area was still known for its highly diverse lagg vegetation. Due to industrial peat extraction and extensive reclamation, within the 20th century, the site underwent massive changes. Ditches and peat cutting have led to strong peat degradation and drying out of the peat surface, with concomitant changes in vegetation. The site has thus a predominance of purple moor-grass (*Molinia caerulea)* and dwarf shrubs (*Calluna vulgaris*, *Erica tetralix*), as well as an abundance of *Betula* spp., whereas *Sphagnum* mosses are mostly absent. The WTL is approximately 43–50 cm below the surface, with a pH of around 4.3, reflecting the site’s altered hydrological and chemical conditions.

##### Pürgschachen and Pichlmeier Moor

Pürgschachen Moor (PU) and Pichlmeier Moor (PI) are two peat bogs located in the Austrian Enns valley in the eastern Alps. The bog PU covers an area of 62 ha and is regarded as the “largest largely intact valley peat bog in Austria with a continuous peat moss cover”.^43^ PU is characterized by its treeless central area and native peatland vegetation, including species such as *Eriophorum vaginatum*, *Rhynchospora alba*, *Pinus mugo*, and *Calluna vulgaris* and S*phagnum* species, such as *S. capillifolium* and *S. divinum/medium*. The water table depth at PU is approximately 17 cm below the surface, with a pH of around 4.12.

PI is located about 5 km east of PU and has undergone numerous anthropogenic interventions, such as peat cutting and drainage. The drainage system is still effective in the eastern side of the bog, though it has not been actively maintained since the 1990s. The north-western part of the bog, a former peat-cutting site shows a natural vegetation succession with *C. vulgaris*, *Vaccinium vitis-idaea*, *Betula pubescens*, *P. mugo*, and *Sphagnum* species such as *S. capillifolium*, *S. divinum/medium*, and *S. fallax* covering the peat extraction pits.^43^^,^^44^ The WTL at PI is approximately −7 cm below the surface, with a pH of around 4.23.

##### Bagno Kusowo

Bagno Kusowo bog (BK) is located within a Natura 2000 site in northern Poland. The bog is a nature reserve and is part of the “Lake Jeziora Szczecineckie” Special Area of Conservation (SAC) Natura 2000. Around 100 years ago, Bagno Kusowo was drained, and peat was extracted while this area was part of Prussia. After World War II the site was again drained. According to Nowakowska et al.,^45^ peat extraction occurred in the bog’s southern part. These peat pits are currently being restored (BK-ER). Despite drainage in the southern part, the northern part remained waterlogged and retains most of its natural ecological characteristics (BK-N). This part was the only one to stay open, and its treeless surfaces support scattered, stunted pine growth. The bog mosses comprise *S. cuspidatum*, *S. divinum/medium*, *S. fallax*, *S. angustifolium*; *Baeothryon caespitosum*, *Drosera rotundifolia*, and *E. vaginatum* are the dominant vascular species growing in the lawn microforms.^46^ At BK-N and BK-ER the average WTL is 11 cm and 18 cm below the surface, respectively, with a pH ranging from 3.3–4.2.

#### Sample analysis

**Figure 1:**
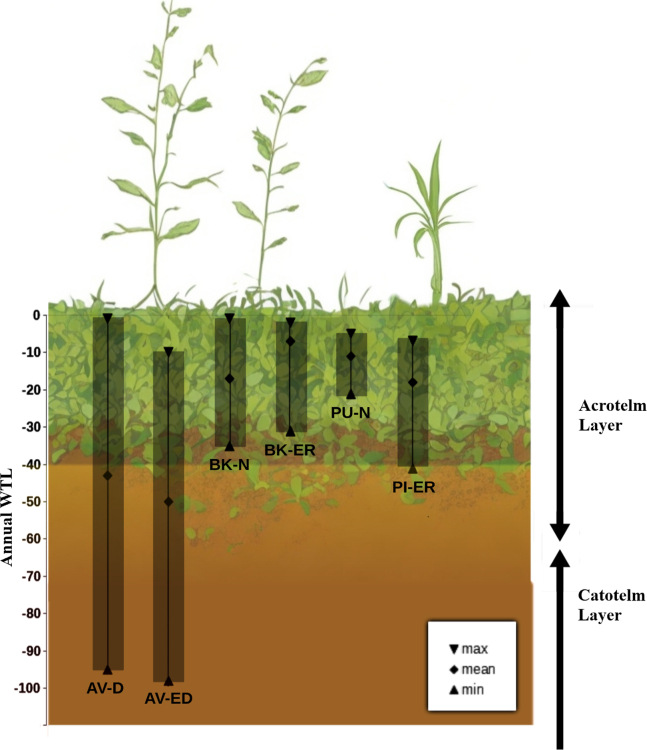
Annual water table level (WTL) at six study sites (AV-D, AV-ED, BK-N, BK-ER, PU-N, and PI-ER) The figure shows maximum, mean, and minimum WTL.

Peat cores were collected from the following sites: At Amtsvenn, Germany, a drained (AV-D) and an extracted and drained (AV-ED) site. In Austria, at Pürgschachen Moor, a natural, undisturbed site (PU-N) and at Pichlmaier Moor, an extracted and rewetted site (PI-ER). In Poland, at Bagno Kusowo, a natural site (BK-N) and an extracted and rewetted site (BK-ER) (see Table 1). At each site, one peat core was collected using a Russian peat corer. The cores were then sectioned into 1 cm intervals at depths of 10 cm, 30 cm, and 80 cm. A total of 3 subsamples were collected per site, with each subsample representing a different depth (10 cm, 30 cm, and 80 cm) and spatial location to account for variability within the site. The WTL at each site has been logged for years (Figure 1).

##### Lipid extraction

In preparation for lipid biomarker analysis, peat samples were freeze-dried and ground with mortar and pestle before extraction. Internal standards were added before extraction to correct for recovery efficiency (100 μL each): squalane (CAS-111-01-3), 0.81 μg/mL for the hydrocarbons, and 1-nonadecanol (CAS 1454-84-8), 1000 μg/mL for alcohols. The samples were saponified with 50 mL of potassium hydroxide (6% in methanol) for 2 h at 70°C in the oven, centrifuged multiple times, and then decanted into a separating funnel. We extracted about 2–3 g of dried peat by ultra-sonication (sonic bath) for 15 min using a 3:1 mixture of methanol and dichloromethane. After each step, we centrifuged the sample at 2500 rpm for 5 min, decanted off the clear extract, and poured the resulting solution into the separation funnel. This process was repeated three times until the solution became clear. The sample was shaken after being acidified to pH 1 with 10% hydrochloric acid and by adding about 40 mL of water. Two phases were clearly visible, the organic phase was cleaned by running through a filter with glass wool and sodium sulfate (NaSO_4_) (CAS-7757-82-6). All the extracts were combined and concentrated on a rotary evaporator under reduced pressure before transferring them to a small vial. The remaining solvent was evaporated under a nitrogen stream to dryness, and the solid sample residue was weighed. As the samples still contained asphaltenes, they were diluted with hexane and filtered again through a glass wool and NaSO_4_ filter several times. In this step, the maltenes were separated from the asphaltenes. The maltenes were further separated into the final fractions of hydrocarbons and alcohols fractions.

The maltene-fraction as mobile phase was applied on an aminopropyl column as stationary phase. The fractions were eluted with hexane for the *n*-alkanes and with dichloromethane and acetone (9:1) for the alcohol fraction. The alcohol fraction was derivatized with 100 μL of BSTFA (N,O-bis(trimethylsilyl)trifluoroacetamide) at 70°C for 1 h to improve volatility and detectability.

We used a GC-MS Agilent 7890A combined with 5975-MSD quadrupole mass spectrometer to identify and quantify *n*-alkanes. The GC was fitted with an HP-5MS ultra inert capillary column (30 m length × 0.25 mm) with a film thickness of 0.25 μm. Helium with 99.9% purity was used as a carrier gas. The sample was injected in splitless mode setting the injector temperature at 310°C. The oven temperature was initially at 60°C and held for 1 min, then ramped to 150°C at a rate of 10°C/min. Finally, we ramped to 325°C at a rate of 4°C/min and kept the temperature constant for 35 min. The compounds were identified by comparing their retention times and mass spectra to those of reference compounds.

#### *n*-alkane molecular indices

Several indices derived from *n*-alkanes provide valuable insights into peat composition. The CPI helps determine whether the origin of peat organic matter is from microorganisms, mosses, or higher terrestrial plants. The ACL reflects changes in species composition and can differentiate between plants that thrive in drier versus wetter environments. Additionally, the plant’s aquatic macrophytes (P_aq_) index, along with the C_23_/C_29_ and C_25_/C_29_ ratios, indicates the relative contributions of aquatic/submerged plants versus vascular plants.^40^

##### CPI

The CPI measures the predominance of odd-numbered *n*-alkanes over even-numbered ones.^47^ As noted, odd carbon chains are indicative of fresh plant organic matter, while even-numbered *n*-alkanes are typically associated with degradation products. CPI values show the difference in peat samples depending on the origin of the peat organic matter and the extent of alternation of the lipid biomarkers.^29^ Higher CPI values suggest a significant contribution from plants and indicate that the organic matter is well preserved. In contrast, lower CPI values point to either a greater abundance of microorganisms or increased degradation.^33^ We calculated the CPI values of the extracted long-chain *n*-alkanes using the modified formula.^48^

(Equation 1) $$\text{CPI}\;n-\text{alkane}=\frac{2\times \left(C_{23}+C_{25}+C_{27}+C_{29}+C_{31}\right)}{\left(C_{22}+C_{24}+C_{26}+C_{28}+C_{30}\right)+\left(C_{24}+C_{26}+C_{28}+C_{30}+C_{32}\right)}$$

##### ACL

The ACL is calculated following Ficken et al.,^48^ and can indicate the source of the organic matter; it describes the average number of carbon atoms per molecule based on the abundance of the odd-carbon-numbered higher plant *n*-alkanes.^49^

(Equation 2) $$\text{ACL}=\frac{C_{23}\times C_{23}+25\times C_{25}+{27\times C}_{27}+29\times C_{29}+31\times C_{31}+33\times C_{33}}{C_{23}+C_{25}+C_{27}+C_{29}+C_{31}+C_{33}}$$

The distribution of *n*-alkanes reflects the response of vegetation types to shifting climate conditions. In drier environments, vascular plants produce longer-chain *n*-alkanes (C_27_ to C_35_), while cooler climates favor submerged plants like mosses, associated with shorter-chain *n*-alkanes (C_23_ to C_25_).^50^ The distribution of *n-*alkanes reflects broader patterns of plant adaptation to climate across different ecosystems, including peatlands. Although the ACL reflects shifts in vegetation type, which are often related to climate changes but can also be influenced by other factors, it does not clarify the specific nature of these climatic changes. However, higher ACL values generally indicate warmer climates, whereas lower ACL values suggest a cooler climate.^22^

##### P_aq_ and further ratios reflecting vegetation composition

Ficken et al.^35^ introduced the P_aq_, an indicator of the relative proportion of aquatic (submerged and floating) macrophytes compared to terrestrial vascular plants based on the fact that C_23_ and C_25_ alkanes are abundant in submerged, floating macrophytes and *Sphagnum* spp*.,* whereas the presence of C_29_ and C_31_ alkanes reflects more terrestrial plant input.^51^

(Equation 3) $$P_{aq}=\frac{\left(C_{23}+C_{25}\right)}{\left(C_{23}+C_{25}+C_{29}+C_{31}\right)}$$

The distribution of *n-*alkanes in aquatic macrophytes is very similar to that of *Sphagnum*, P_aq_ values may thus indicate the dominance of *Sphagnum* species over other peat-forming plants,^52^^,^^53^ associating higher P_aq_ values with bog peat primarily composed of S*phagnum* spp.^54^ Terrestrial plants typically exhibit P_aq_ values below 0.1, emergent macrophytes range between 0.1 and 0.4, and fresh *Sphagnum* is characterized by values exceeding 0.4 in peatland settings. Although most *Sphagnum* species lack C_29_, a few reach their maximum at C_31_, showing the complexity of *Sphagnum* biomarker signatures.^23^^,^^37^^,^^55^ The inclusion of ratios such as C_23_/C_29_ and C_25_/C_29_ alongside P_aq_ allows for a more direct comparison between different *Sphagnum* and vascular plant species.

Zhang et al.^50^ noted that *Sphagnum* species that prefer cold and wetter conditions (Hollow species) have a maximum at C_23_, while those preferring warm and drier conditions (Hummock species) peak at C_25._ The variation in the *n*-alkane profiles between wet and dry *Sphagnum* species thus helps to distinguish between the species based on their preferred moisture level. The C_23_/C_25_ ratio can be used as an indicator of moisture conditions, as it has been previously applied in peatland studies to evaluate changes in *Sphagnum* species composition.^56^

All these indices are based on the specific *n*-alkane distribution patterns of certain species, as described previously. It is important to note that these *n*-alkane indices are only relative measures, and none of them are standardized. Variation in values and formulas of *n*-alkanes indices can differ across different studies depending on factors, such as the ecosystem, vegetation, and climate.^29^^,^^48^

## Results

### Concentration of *n*-alkanes

**Figure 2:**
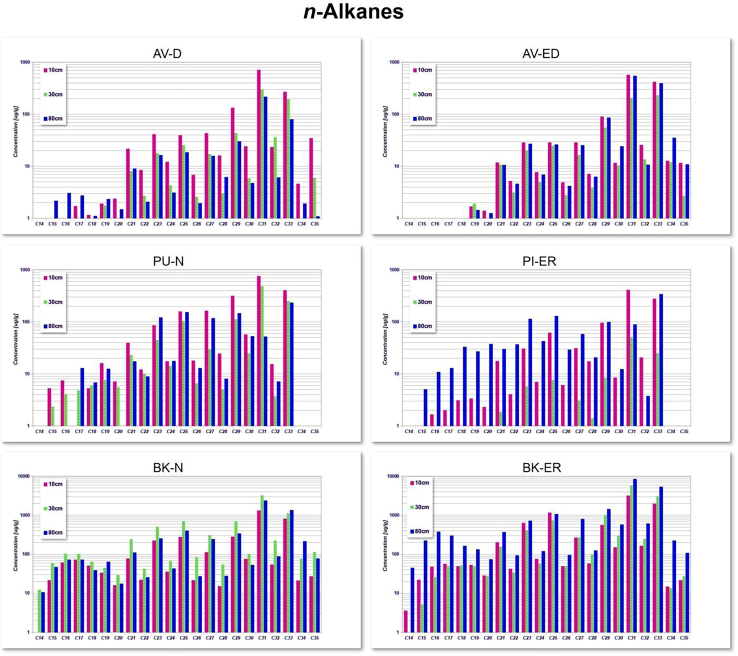
Distribution of *n*-alkane (C_14_–C_35_) across six sites (AV-D, AV-ED, BK-N, BK-ER, PU-N, and PI-ER) at depths of 10 cm, 30 cm, and 80 cm Bars indicate depth-specific variations in concentrations (μg/g, log scale).

In this study, we analyzed *n*-alkanes with carbon chain lengths ranging from C_14_ to C_35_. We measured the concentrations of these *n*-alkanes at different depths across all study sites, as illustrated in Figure 2. The raw data for the *n*-alkane concentrations across all sites and depths is available in the Table S1A.

Our investigation reveals a distinct distribution pattern of *n*-alkanes across all the sites. In general, the shorter chain *n*-alkanes (C_17_, C_18_, and C_19_) are less abundant, whereas the medium chain *n*-alkanes (C_23_ and C_25_) are more abundant, and In particular, the longer-chain *n*-alkanes (C_27_–C_35_) showed highest abundance.

The shorter-chain *n*-alkanes (C_15_–C_20_) are present in lower concentration at the AV-D and AV-ED sites compared to PU-N and PI-ER, where they reach their highest concentrations at deeper depths. In contrast, the BK-N and BK-ER sites exhibit the highest fraction of short-chain *n*-alkanes, with a maximum at C_17_, particularly at depths of 30 cm and 80 cm.

The highest concentration of mid-chain *n*-alkanes (C_23_–C_25_) is observed at 10 cm depth at the AV-D site, ranging from 39 to 41 μg/g with an average ($\bar{x}$) 40 μg/g. At the AV-ED site, the concentrations range from 28 to 29 μg/g ($\bar{x}$: 29 μg/g), with the highest peak observed for C_23_. In contrast, at the PU-N and PI-ER sites, the highest concentrations of mid-chain n-alkanes (C_23_–C_25_) are observed at 80 cm depth. At PU-N site, concentration range from 120 to 153 μg/g ($\bar{x}$: 137 μg/g), while at PI-ER site, they range from 113 to 129 μg/g ($\bar{x}$: 121 μg/g), with again C_23_ showing the highest peak at both sites. At the BK-N site, the highest concentration occurs at a depth of 30 cm, ranging from 502 to 688 μg/g ($\bar{x}$: 595 μg/g). Comparatively, at BK-ER site, the concentrations are highest at 80 cm depth, with the values ranging from 715 to 1076 μg/g ($\bar{x}$: 896 μg/g). These variations in the distribution of mid-chain *n*-alkanes at different depths suggest different contributions of organic matter or varying decomposition processes across the sites.

At the AV-D and AV-ED sites, the concentration of long-chain *n*-alkanes (C_27_–C_35_) decreases with depth of 10 cm. At AV-D, values range from 34 to 711 μg/g ($\bar{x}$: 238 μg/g), while at AV-ED, they range from 12 to 566 μg/g ($\bar{x}$: 222 μg/g). Similarly, at the PU-N and PI-ER sites, the highest concentrations of long-chain *n*-alkanes are also found at 10 cm depth. At PU-N values range from 162 to 755 μg/g ($\bar{x}$: 327 μg/g), whereas at PI-ER, they range from 31 to 408 μg/g ($\bar{x}$: 162 μg/g). At the BK-N and BK-ER sites, the maximum concentrations of long-chain *n*-alkanes are observed at different depths. At BK-N, the highest values are observed at 30 cm, ranging from 113 to 3,260 μg/g ($\bar{x}$ = 1,099 μg/g), while at BK-ER, the maximum concentration is found at 80 cm, with values ranging from 107 to 8,426 μg/g ($\bar{x}$ = 3,236 μg/g).

#### Carbon preference index and average chain length

**Figure 3:**
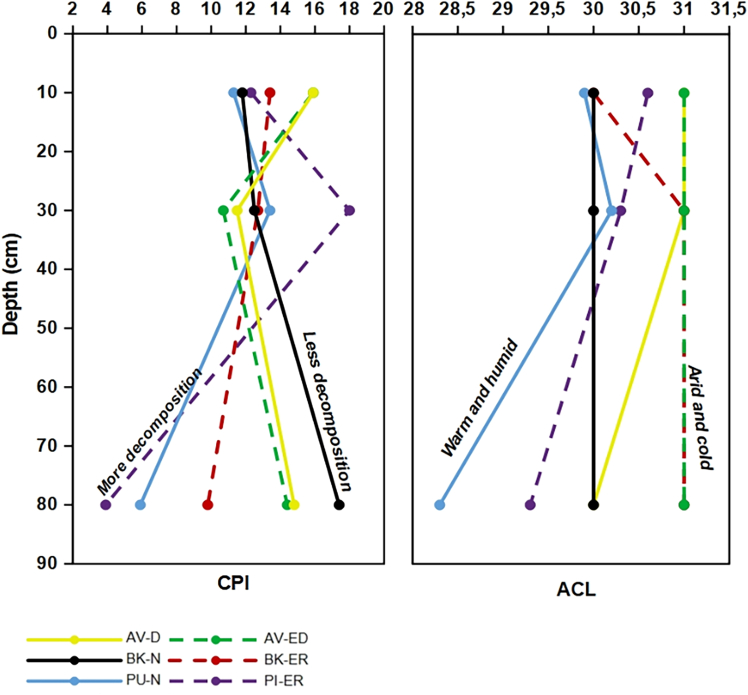
Carbon preference index (CPI) and average chain length (ACL) of peat samples across six sites (AV-D, AV-ED, BK-N, BK-ER, PU-N, and PI-ER) at depths of 10, 30, and 80 cm

The CPI and ACL values vary distinctively across different sites and depths, as shown in Figure 3.

The CPI values show a similar trend at both the AV-D and AV-ED sites, with the highest values observed at different depths. At AV-D the highest CPI value 14.1 is observed at a depth of 80 cm, while at AV-ED, the highest and CPI value 15.9 is observed at 10 cm. At the PU-N and PI-ER sites, the highest CPI values 13.4 and 18.0, respectively, are observed at a 30 cm depth, decreasing with increasing depth. Both sites show high decomposition with the lowest CPI values observed at a depth of 80 cm. In contrast, at the BK-N site, the CPI increases with depth reaching its highest value of 17.4 at 80 cm. In contrast at BK-ER the CPI value decreases with depth, with the highest value of 13.4 observed at 10 cm depth.

At the AV-D and AV-ED sites, there is a minor change in the dominant vegetation at a depth of 80 cm, as indicated by a decrease in the ACL from 31 to 30. At the PU-N and PI-ER site, a shift in the plant species composition is suggested by the decrease in ACL at 80 cm. Nevertheless, it appears that there has not been a drastic/major change in the flora at the BK-N and BK-ER sites as the ACL remains consistently around 30 in the BK-N and increases slightly in the BK-ER site.

#### P_aq_ and other vegetation ratios

**Figure 4:**
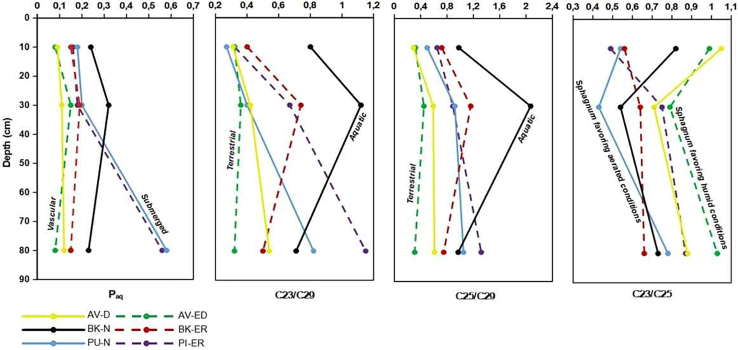
P_aq_ index and C_23_/C_29_, C_25_/C_29_, and C_23_/C_25_ ratios of peat samples across six sites (AV-D, AV-ED, BK-N, BK-ER, PU-N, and PI-ER) at depths of 10, 30, and 80 cm

The variations in P_aq_ values and other related indices across different sites and depths are illustrated in Figure 4. At the AV-D and AV-ED sites, P_aq_ values are below 0.1 at the shallower depth (10 cm), increasing at 30 cm depth before decreasing again. The ratios of *Sphagnum* to vascular plants C_23_/C_29_ and C_25_/C_29_ show a slight increase with depth at the AV-D site. At the AV-ED site, these ratios follow a similar pattern, with a slight increase at 30 cm depth. The C_23_/C_25_ ratio also follows a similar pattern at both bog sites, showing a slight decrease at 30 cm depth.

Conversely, at the PU-N and PI-ER sites, P_aq_ values are greater than 0.1, with values greater than 0.5 observed in the deepest layers (80 cm), indicating a strong preference for aquatic vegetation at these depths. In addition, the ratio of *Sphagnum* to vascular plants, specifically the C_23_/C_29_ and C_25_/C_29_ ratios shows a similar trend at both sites, indicating a greater contribution of *Sphagnum* relative to vascular plant input. The C_23_/C_25_ ratio also follows a similar trend, reflecting a greater contribution of *Sphagna* which prefer wetter conditions at the 80 cm depth, compared to vascular plants.

Similarly, at the BK-N and BK-ER sites, P_aq_ values are above 0.1 with the highest values observed at 10 cm, suggesting enhancing aquatic vegetation at these shallow depths. The ratios of C_23_/C_29_ and C_25_/C_29_ for *Sphagnum* to vascular plants increase at 10 cm at both sites, following a similar trend, with a slight increase at 80 cm. The C_23_/C_25_ ratio indicates that *Sphagnum* species preferring drier conditions are generally dominant over those that prefer wetter conditions.

### Concentration of *n*-alkanols

**Figure 5:**
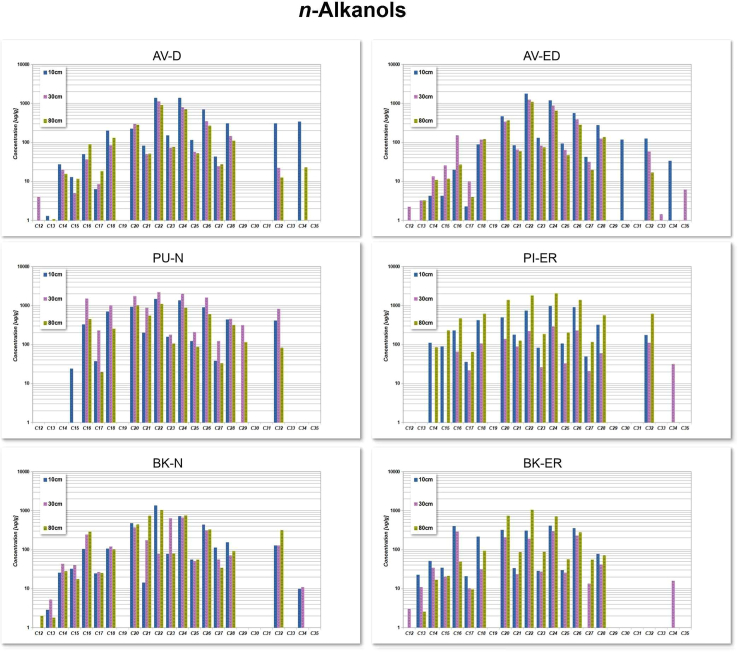
Distribution of *n*-alkanols (C_12_–C_35_) across six sites (AV-D, AV-ED, BK-N, BK-ER, PU-N, and PI-ER) at depths of 10 cm, 30 cm, and 80 cm Bars indicate depth-specific variations in concentrations (μg/g, log scale).

The *n*-alkanol fractions isolated from the study sites have carbon chain lengths from C_12_ to C_34_, with a strong predominance of even-over-odd carbon numbers as illustrated in Figure 5. The raw data for the *n*-alkane concentrations across all sites and depths is available in the Table S1B.

Short-chain *n*-alkanols (C_15_ to C_18_) are abundant across all the peat bog sites, while mid-chain *n*-alkanols (C_20_ to C_26_) are dominant at all the study sites. At the AV-D and AV-ED sites, mid-chain *n*-alkanols peak at C_22_, with the highest concentrations observed at a depth of 10 cm. At AV-D, concentration range from 1,375 to 1,382 μg/g ($\bar{x}$ = 1,378 μg/g), while at AV-ED, they range from 1,182 to 1,765 μg/g ($\bar{x}$ = 1,473 μg/g). At the PU-N and PI-ER sites, mid-chain *n*-alkanols also peak at C_22,_ with the highest concentrations observed at 30 cm and 80 cm. At PU- N, concentration range from 1953 to 2169 μg/g ($\bar{x}$: 2061 μg/g) at 10 cm depth, while at PI-ER they range from 1807 to 2028 μg/g ($\bar{x}$: 1917 μg/g) at 80 cm depth. Similarly, at the BK-N and BK-ER sites, peak concentrations of mid-chain *n*-alkanols are observed at depths of 10 cm and 80 cm, respectively. At BK-N site, concentrations range from 721 to 1,342 μg/g ($\bar{x}$ = 1,031 μg/g), while at BK-ER, they range from 700 to 1,051 μg/g ($\bar{x}$ = 875 μg/g).

Conversely, long-chain *n*-alkanols (C_29_ to C_36_) are present at low levels at all sampled sites. Specifically, C_29_ is not detected at almost all sites except for PU-N, where it occurred at depths of 30 cm and 80 cm. Additionally, C_31_ is not detectable across all sites.

### Steroids and pentacyclic triterpenoids

At our study sites, the distribution of steroids and pentacyclic triterpenoids in the peat varies considerably between samples and through each peat core (Table 2).

**Table 2:** Concentration of steroids and pentacyclic triterpenoids (μg/g) across six sites (AV-D, AV-ED, BK-N, BK-ER, PU-N, and PI-ER) at depths of 10, 30, and 80 cm

Site	AV-D			AV-ED			PU-N			PI-ER			BK-N			BK-ER		
Depth (cm)	10	30	80	10	30	80	10	30	80	10	30	80	10	30	80	10	30	80

**Steroids (concentration (ug/g])**																		

Brassicasterol	**71**	18	13	25	27	**42**	187	**203**	130	104	34	**296**	51	72	**87**	81	**83**	66
Campesterol	**342**	111	60	**97**	72	36	896	748	**994**	352	191	**2070**	195	262	**442**	346	69	**379**
Stigmasterol	**308**	198	47	86	**189**	54	**900**	543	789	348	246	**1425**	132	78	**188**	**330**	40	241
Sitosterol	**895**	573	429	386	367	**453**	4670	5317	**6641**	1533	1290	**13598**	1462	837	**2656**	**1845**	766	1247
Stigmastanol	**208**	171	150	164	164	**212**	782	701	**1998**	198	258	**4855**	95	76	**327**	109	**165**	148
Lanosterol	–	–	**88**	–	–	–	**165**	–	0	0	0	0	–	–	–	–	–	–
Ergosterol	–	–	–	–	–	–	82	**116**	73	**171**	4	0	–	**12**	–	–	–	–
Campestanol	–	–	–	–	–	–	148	187	**354**	28	42	**1099**	–	–	–	–	–	–
Cholestanol	–	–	–	–	–	–	51	**114**	80	14	6	**241**	–	–	–	–	–	–
Cholesterol	–	–	–	–	–	–	–	–	–	–	–	–	–	–	–	–	**71**	–

**Pentacyclic triterpenoids concentration (ug/g)**																		

Taraxera-14-en-3b-ol	**87**	42	37	74	66	**81**	**350**	264	79	**71**	23	–	**125**	121	55	0	0	**177**
Cycloartenol	**240**	227	104	127	**145**	49	614	**848**	682	398	178	**1553**	**540**	152	247	**322**	257	109
24-Methylcycloartenol	**759**	375	114	**381**	237	118	1120	**1943**	1862	633	346	**3802**	**980**	494	688	**439**	285	119
α-Amyrin	**896**	0	0	**38**	0	0	–	–	–	–	–	–	–	**46**	0	0	0	**491**
β-Amyrin	–	–	–	–	–	–	–	**527**	–	–	–	–	–	–	–	–	–	–
Acyclic biphyten diol	–	0	**20**	0	–	0	36	16	**155**	–	–	**535**	–	0	0	0	0	0
Taraxera-14-ene	**515**	131	60	**353**	252	300	**1432**	321	1022	**512**	40	455	161	**1726**	589	0	0	0
Taraxera-XX-ene	**5**	1	2	1	**2**	1	**26**	14	11	**25**	3	7	12	**55**	2	0	0	0
Oleane-12-ene	**33**	15	17	25	**28**	40	**165**	89	108	**220**	17	50	77	**322**	41	0	0	0
Friedeooleane-7-ene	**29**	2	0	**6**	0	0	**124**	20	–	**65**	2	44	0	**148**	0	0	0	0

At the AV-D site, sitosterol shows the highest concentration at a depth of 10 cm, followed by campesterol, stigmasterol, and its degradation product, stigmastanol. At the AV-ED site, sitosterol is most concentrated at a depth of 80 cm, with stigmastanol as the second highest. Conversely, at the PU-N and PI-ER sites the concentration of steroids is highest at a depth of 80 cm with sitosterol being the most abundant, followed by its degradation product stigmastanol and campesterol. At the BK-N and BK-ER sites, sitosterol dominates at all measured depths, with the highest concentration observed at 80 cm in BK-N and 10 cm in BK-ER. The second-highest concentrations are those of campesterol at both sites. Lanosterol is found at AV-D and PU-N at depths of 80 cm and 10 cm, respectively. Ergosterol has been identified at PU-N and PI-ER, as well as at a depth of 30 cm at BK-N.

Pentacyclic triterpenoids (PCTs) are one of the most important classes of secondary plant metabolites.^57^^,^^58^ Pentacyclic triterpenoids with lupane, oleanane, ursane, and friedelane are considered biomarkers of angiosperms.^22^^,^^59^^,^^60^

At the AV-D and AV-ED sites, pentacyclic triterpenoids (PCTs) are present at all depths, with the highest concentrations observed at a depth of 10 cm at both sites. Notably, AV-D site has the highest concentration of α-amyrin at this depth, whereas AV-ED site has the lowest concentration of this compound. This pattern suggests that PCTs derived from vascular plants are typically more concentrated in the upper layers, indicating a greater contribution of woody plants to the peat at these sites. At the PU-N site, compounds such as cycloartenol, 24-methylcycloartenol, and β-amyrin show increased concentrations at a depth of 30 cm. In contrast, at the PI-ER site, these compounds peak at 80 cm depth except for β-amyrin, which is completely absent. Similarly, taraxera-14-ene, oleane-12-ene, and friedoleane-7-ene reach their highest levels at a depth of 10 cm at both sites, indicating a higher abundance of shrubs as well as vascular plants at these sites.

The BK-N site shows a higher concentration of PCTs at all the depths compared to the BK-ER site where these compounds are less abundant. Both sites show the highest concentrations of 24-methylcycloartenol and cycloartenol at a depth of 10 cm. Additionally, compounds like taraxerine-14-ene, oleane-12-ene, and friedoleane-7-ene peaks at 30 cm in BK-N but are completely absent in BK-ER. Conversely, α-amyrin is not detected at BK-N but reaches its highest concentration at 80 cm in BK-ER indicating a significant contribution of shrubs at the BK-ER site.

### Bacterial-derived biomarkers (hopanoids)

**Figure 6:**
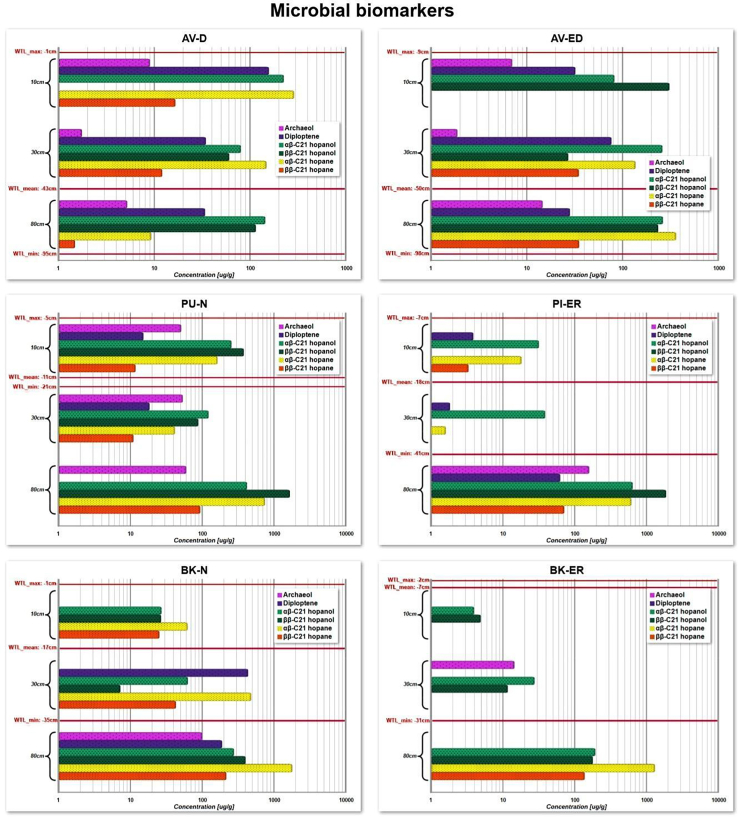
Concentration of hopanoids and archaeol in peat samples across six sites (AV-D, AV-ED, BK-N, BK-ER, PU-N, and PI-ER) at depths of 10, 30, and 80 cm Bars indicate depth-specific variations in concentrations (μg/g, log scale). The red horizontal lines indicate water table levels, with maximum, mean, and minimum water table levels.

The concentration of hopanoid biomarkers and archaea at each study site is shown in Figure 6. At the AV-D site, the hopanoids concentration varies with depth. At 10 cm depth, 17α(H),21β(H)-hopane is relatively abundant. By 30 cm, most hopanoid concentrations decrease, but 17α(H),21β(H)-hopane remains the highest. At 80 cm depth, there is a further reduction in most of the biomarkers; however, 17α(H),21β(H)-hopanol shows a notable increase. In contrast, at the AV-ED site, the highest concentration of 17β(H),21β(H)-hopanol is observed at 10 cm. At 30 cm depth, there is an overall increase in hopanoid concentration, with 17α(H),21β(H)-hopanol reaching its highest peak. At 80 cm, hopanoid concentration increases significantly, particularly for 17α(H),21β(H)-hopane, which shows a notable spike. The high abundance of 17α(H),21β(H)-hopanol at shallower depths on both sites suggests early isomerization of 17β(H),21β(H)-hopane through diagenesis, probably related to the acidic peat environment.^61^^,^^62^^,^^63^ Diploptene is found in both sites, with the highest concentrations observed at 10 cm depth at AV-D and at 30 cm depth at AV-ED.

At the PU-N site, the concentration of hopanoid biomarkers varies at different depths. At 10 cm 17β,21β(H)-21-hopanol is the most abundant species, while at 30 cm, the concentrations of all hopanoids slightly decrease. Notably, at 80 cm there is a significant increase in 17β(H),21β(H)-21-hopanol indicating the presence of aerobic methanotrophic bacteria or indicating favorable preservation conditions.^64^ Comparatively, the PI-ER site shows the lowest hopanoid concentration, with 17β(H),21β(H)-21-hopanol being completely absent at 10 and 30 cm depths. In contrast to the 80 cm, which shows the highest concentration of 17β(H),21β(H)-21-hopanol. Moreover, the biogenic hopanoids 17β(H),21β(H)-22-hopanol and 17β,21β(H)-21-hopane are found in the highest concentrations at the depth of 80 cm within the anoxic catotelm of both bogs and are mainly synthesized by aerobic bacteria. However, the occurrence of aerobic methanotrophic bacteria has been reported also in anaerobic peat layers.^65^^,^^66^^,^^67^ Additionally, diploptene is present at low concentrations on both sites, with the highest concentration observed at 30 cm at the PU-N site and at 80 cm at PI-ER site.

At the BK-N site, hopanoid biomarker concentrations vary, with depth, with 17α(H),21β(H)-22- hopane being the most abundant across all depths with the highest concentration at 80 cm. At 30 cm, the concentration of most of the hopanoids decreases except for diploptene, which shows the highest concentration. Comparatively, the BK-ER shows a low abundance of hopanoids, with concentrations hardly detectable at 10 and 30 cm depth. However, at 80 cm depth, there is a significant increase in the concentration of 17α(H),21β(H)-21-hopane, suggesting contributions from various bacteria, particularly aerobic bacteria, at this depth.^68^^,^^69^ In this peat core, hopanoid biomarkers are highest in permanently waterlogged conditions, but also show abundance at 30 cm depth, located between the acrotelm and catotelm. Additionally, BK-N contains diploptene at 30 and 80 cm depths in contrast to BK-ER, where this biomarker is completely absent.

#### Archaeol

At the AV-D and AV-ED sites, despite the dry conditions, archaeol is present in all samples, with the highest concentration observed at depths of 10 and 80 cm. This suggests that archaeol is situated below the average water table. In contrast, at the PU-N site, archaeol is present in all samples, with the highest concentration found at 80 cm depth. At the PI-ER site, archaeol is only well detected at 80 cm depth, where the water table is around 9 cm, creating the anoxic conditions favorable for archaea. Conversely, at the BK-N and BK-ER sites, archaeol is absent at both 10 and 30 cm depths but appears at 80 cm depth at the BK-N site and at 30 cm depth in the BK-ER site, below the minimum WTL of −35 cm, which is consistent with its requirement for anaerobic conditions.

## Discussion

### Composition and origins of lipid biomarkers in peat bogs

#### *n*-alkanes

The lower percentages of short-chain *n*-alkanes at the AV-D and AV-ED sites indicate lower contribution from sources of short chain *n*-alkanes potentially due to drier conditions and lower water table, which limit the growth of aquatic vegetation. While the higher concentrations at PU-N and PI-ER suggest additional retention of organic material, potentially derived from algal sources. In contrast, the BK-N and BK-ER sites exhibit the highest percentages of short-chain *n*-alkanes. Their presence can suggest that the organic matter in the peat has notable contributions from bacteria, algae, or aquatic plants.^70^ The wetter, more waterlogged conditions could promote microbial activity and algal productivity, enhancing the preservation and accumulation of short-chain *n*-alkanes at these sites.

All the samples from our study sites, from near-natural to degraded and restored, show higher concentrations of mid-chain *n*-alkanes (C_23_– C_25_). The presence of mid-chain *n*-alkanes (C_23_ and C_25_) alkanes is a characteristic feature of *Sphagnum* species, as reported by Nichols et al.^52^; Nott et al.^71^; Pancost et al.^23^; Skreczko et al.^11^ In our study sites, mid-chain *n*-alkanes observed at shallower depths suggest that anaerobic conditions and the presence of specific vegetation, such as *Sphagnum* mosses are particularly favorable for their accumulation or preservation that are typically found in waterlogged areas. Additionally, environmental factors like temperature, humidity, and seasonal rainfall variations likely influence vegetation types and litter quality, indirectly affecting mid-chain *n*-alkane inputs. Conversely, deeper layers are more conducive to the preservation or concentration of these compounds, potentially influenced by different depositional conditions or changes in vegetation over time. These variations in the distribution of mid-chain *n*-alkanes at different depths suggest different contributions of organic matter or varying decomposition processes across the sites.

Our study sites also show higher concentrations of long-chain *n*-alkanes, which could be related to the abundance of vascular plants. Notably, each sampled site showed elevated levels of long-chain *n*-alkanes especially C_31_ whereas long-chain homologues (usually > C_21_) are typically indicative of vascular plant origin.^72^ Furthermore, studies generally attribute the presence of higher molecular weight *n*-alkanes (C_29_, C_31_, and C_33_) in vascular plants to their waxy cuticular origin.^28^^,^^73^ Indeed, due to partially fluctuating, unstable hydrology, most of the sites under study harbor vascular plants, such as sedges and dwarf shrubs, and this was accordingly reflected in *n*-alkanes.

This distribution pattern of long chain *n*-alkanes is consistent with the dominant hydrocarbon profiles observed in species inhabiting different peatland sites. For example, *Rhynchospora alba* and the leaves of *Calluna vulgaris* synthesize high amounts of C_31_; *Molinia caerulea*, or the stem of *Calluna*, show peaks at C_29_; or *Drosera* sp. at C_27_.^74^ Moreover, researchers have observed higher concentrations of C_31_ in certain *Sphagnum* species such as *S. affine*, *S. capillifolium*, *S. medium/divinum*,^48^ and *S. teres*.^75^

Our study sites show significantly higher concentrations of C_31_, which may differ from those reported by Baas et al.^37^ and Nichols et al.^52^ These studies identified long-chain *n-*alkanes (C_23_ to C_31_) in specific *Sphagnum* species and other bog-forming plants. The higher C_31_ concentrations observed in our study may be attributed to the combined contributions of both vascular plants and *Sphagnum* species in peat samples, whereas previous studies primarily focused on specific plant species. Based on the distribution observed in this study, it can be inferred that the organic matter at the studied sites originated from different sources, such as microbial biomass, *Sphagnum*, or vascular plants.^76^ However, further research is needed to explore the potential influence of additional factors on these observed differences.

In conclusion, while *Sphagnum* and vascular plants (e.g., *Eriophorum vaginatum*) are key contributors to organic matter in peatlands, other sources also play significant roles as apparently reflected in the biomarker profiles. Microbial biomass from bacteria and fungi enhances nutrient cycling, and surrounding aquatic plants and algae enhance organic inputs. The diversity of organic matter sources highlights the complex interactions within peatland ecosystems. The *n*-alkane profiles not only serve as biomarkers for interpreting the primary sources of organic matter but also provide insights into the historical dynamics of vegetation and environmental conditions.

#### *n*-Alkanols

In contrast to *n*-alkanes, *n*-alkanols are considered to undergo more rapid post-depositional degradation.^77^ *n*-alkanols are one of the important biomarker compounds, derived predominantly from the epicuticular waxes of vascular plants.^78^ The observed *n*-alkanol distribution at all three sites, with a primary peak at C_22_, C_24_, and C_26_, is indicative of organic matter derived from microorganisms and vascular plants, respectively.^29^^,^^79^ Our results align with the *n*-alkanol profiles reported by Baas et al.,^37^ which indicate that C_24_ and C_26_ are typically more abundant than other *n*-alkanols. Similarly, Ficken et al.^48^ also found that C_24_ and C_26_ alcohols were the most abundant in two different species of *Sphagna* providing further evidence of plant input.

In contrast to the predominance of vascular plant waxes in *n*-alkane distributions, the major sources of *n*-alkanols vary by chain length.^80^ Identifying the source organisms of *n*-alkanols is challenging due to their potential biochemical or geochemical origins. For example, C_16_–C_24_ alkanols can be derived from algae or bacteria, C_22_–C_24_ alkanols from submerged plants, and C_22_–C_28_ alkanols from either submerged plants, emergent plants, or terrestrial plants.^48^^,^^81^^,^^82^ The diversity of potential sources for *n*-alkanols complicates the interpretation of the results. In addition, *n*-alkanols exhibit lower recalcitrance than alkanes and are selectively broken down by microorganisms,^83^ adding further complexity to their interpretation as biomarkers.

Plant-derived *n*-alkanols, in contrast to *n*-alkanes, show a clear predominance of even over odd carbon numbers.^22^^,^^80^ Moreover, the degradation patterns observed in *n*-alkanols suggest that their use as paleoecological proxies provides valuable insights into past ecological conditions.^84^^,^^85^

#### Steroids and plant-derived pentacyclic triterpenoids

Steroids are tetracyclic triterpenoid lipids that are ubiquitous in all eukaryotes.^86^ They can provide important insights into the origin and preservation of organic matter and identify the original vegetation contributions to the peatland.^24^^,^^81^ The major sterols found in our study sites are sitosterol, stigmasterol, campesterol, and brassicasterol, which indicate input from both vascular plants and mosses.^87^ Although sitosterol is commonly used as a biomarker for terrestrial organic matter in geochemical studies, some researchers suggest that it may originate from phytoplankton or aquatic macrophytes.^88^

Our findings are in strong agreement with previous studies on sterols in peat deposits^23^^,^^29^^,^^37^^,^^74^^,^^87^^,^^89^ and confirm the consistency and reliability of sterol profiles as indicators of vegetation and microbial dynamics in peat ecosystems. Sterols, such as stigmasterol and sitosterol, which are are commonly found in many peat-forming plants, including *Sphagnum* as noted in previous studies.^88^^,^^90^^,^^91^ These findings support the higher concentrations of mid-chain *n*-alkanes (C_23_–C_25_) in wetter, waterlogged conditions, further highlighting the significant contributors of *Sphagnum* mosses to the organic matter. Moreover, studies^29^^,^^87^^,^^92^ have demonstrated that microbial hydrogenation of sterols in peat can result in the production of stanols from the original 5-sterols; it is therefore essential to identify either the original sterols or their corresponding degradation products within the same core material.

The stigmasterol and sitosterol are common sterols found in peat.^28^ These sterols play significant roles in indicating plant inputs and the decomposition processes occurring within the peat ecosystem. The presence of lanosterol, a key intermediate in the biosynthesis of sterols in both fungi and animals, provides further ecological insight, as it is converted into ergosterol in fungi.^93^ Ergosterol serves as a biomarker for fungi and certain microalgae and protozoa but not in bacteria, insects, or plants.^94^ It serves as a key biomarker to estimate fungal biomass in plants and soils.^95^ The relative abundance of sterols at different depths reflects the influence of environmental conditions, including past plant inputs and varying sources of peat organic matter. This variability highlights how different ecological factors interact to shape steroid distribution within the peat ecosystem.

In addition to sterols, pentacyclic triterpenoids (PCTs) are one of the most important classes of secondary plant metabolites.^57^^,^^58^ PCTs with lupane, oleanane, ursane, and friedelane are considered as biomarkers of angiosperms.^22^^,^^59^^,^^60^

PCTs, like *α*-amyrin, *β*-amyrin, and cycloartenol were identified in our study sites and are considered precise geochemical markers for vascular plants.^88^^,^^96^^,^^97^ Interestingly, *α*-amyrin and *β*-amyrin have been detected in the roots of *Erica tetralix* and in the leaves of *Calluna vulgaris*^37^^,^^98^ and their presence in our study supports the identification of these specific plant sources. The presence of cycloartenol, a key triterpenoid marker synthesized primarily by plants or algae with its presence indicating plant-based contributions to the peat consistent with findings from previous studies.^99^ Additionally, triterpenoids, mainly taraxer-14-ene and taraxast-20-ene, have been associated with dwarf shrubs, especially their roots.^23^ Dwarf shrubs, primarily *C. vulgaris* and *E. tetralix*, exhibit a varying degree of dominance across all the study sites in our study (Drollinger et al., 2019; Gałka et al., 2017; Lemmens, personal communication).^29^^,^^37^ This is further supported by the *n*-alkane data, which show elevated concentrations of long-chain *n*-alkanes (C_29_ and C_31_**)** across all study sites, indicating that vascular plants and shrubs are key contributors to the organic matter in the peat.

The combined analysis of sterols and PCTs in our study enhances the understanding of the ecological dynamics of peat ecosystems. While sterols offer insights into plant and microbial contributions, their degradation products complicate source identification. Similarly, the presence of PCTs strengthens our understanding of the vascular plant contributions to peat organic matter The variability in biomarker distribution at different depths not only reveals the state of organic matter preservation but also reflects past environmental conditions and the dynamics of plant and microbial activity during peat formation.^60^

### Environmental influences on peat decomposition and degradation

#### Carbon preference index and average chain length

Biomarkers derived from different biological sources exhibit different CPI values, highlighting their utility in tracing organic matter sources within peatlands. According to Zheng et al.^51^ *n*-alkanes from cuticular waxes of vascular plants typically have higher CPI values whereas those derived from bacteria and algae have lower CPI values.

The CPI at the AV-D site is highest at 80 cm whereas, at the AV-ED site, it reaches its maximum at 10 cm. At the AV-ED site, peat harvesting had occurred and the peat at the surface is thus considerably older than the peat at the AV-D site. Despite both the sites being drained and potentially sharing the same water table significant decomposition is evident. The observed variability in CPI values may indicate differences in the preservation of organic matter, likely influenced by climatic conditions that favor vascular plant contributions while reducing bacterial and algal activity, thereby affecting the degree of organic matter alteration.

At the PU-N and PI-ER sites, the CPI reaches its highest value at a depth of 30 cm at both sites, with the most significant decomposition occurring at 80 cm depth. Although these bogs are at some distance from each other and have different water tables, they show a similar trend in decomposition at 80 cm. At the BK-N site, the CPI is highest at 80 cm depth compared to the BK-ER site, which shows the lowest. Nevertheless, despite the differences in water tables, the CPI follows a similar trend and shows no signs of decomposition at either site.

The variation in CPI values observed across these sites is consistent with findings from previous studies. For example, Lehtonen et al.,^90^ observed a decrease in CPI values with depth, indicating increased microbial activity. Conversely, Harrault et al.^100^ found that an increase in CPI values with depth may reflect a temporal shift in vegetation rather than decomposition. The observed variation in CPI values across different sites and depths is influenced by many factors, such as past vegetation and environmental change.

ACL is used to reconstruct climatic changes.^22^ The distribution of *n*-alkanes reflects how different vegetation types respond to shifting climatic changes.^101^ It has been suggested that drier conditions enhance the production of the longer-chain *n*-alkanes in terrestrial plants. In contrast, colder and wetter climates increase the production of medium-chain *n*-alkanes in submerged plants such as certain mosses.^53^^,^^102^ Indeed, ACL values are higher in the upper layers at the AV-D and AV-ED sites compared to those at the PU-N, PI-ER, BK-N, and BK-ER sites. These findings agree with those of Bhattacharya et al.,^40^ who suggested the lower values in the deeper depth reflect a slightly wetter environment, while the higher ACL in the upper layer index reflects drier conditions. Nevertheless, all three sites, with their different conditions, exhibit minor fluctuations in ACL throughout the profiles.

#### P_aq_ and other vegetation ratios

At the AV-D site, the P_aq_ value indicates dry conditions and the ratio of C_23_/C_29_ and C_25_/C_29_ shows a similar trend at 10 cm depth but slightly increases at 80 cm depth, suggesting an increasing contribution of *Sphagnum* mosses relative to vascular plants at greater depths. At AV-ED, the P_aq_ value also indicates dry conditions, with a slight increase in C_23_/C_29_ and C_25_/C_29_ ratios at 30 cm in AV-ED suggesting the minimum contribution of *Sphagnum* at this depth. At 10 cm depth, there is a noticeable influence from vascular plants such as *Molinia caerulea* at the AV-ED site and from dwarf shrubs at the AV-D site. This pattern suggests that the more recent changes in vegetation, characterized by the dominance of vascular plants with minimal abundance of *Sphagnum* species, is already to some extent reflected in the biomarker data. The *Sphagnum* ratio (C_23_/C_25_) increases at 10 cm depth in AV-D and at 80 cm depth in AV-ED, which may suggest a balance between wet and dry-favoring *Sphagnum* is likely responsible.

In contrast, at the PU-N and PI-ER sites, the highest P_aq_ value and ratios of C_23_/C_29_ and C_25_/C_29_ increase with depth, indicating a greater contribution from *Sphagnum* relative to vascular plants in the past. This suggests that deeper peat layers were deposited under wetter conditions, typical of *Sphagnum-*dominated environments. The C_23_/C_25_ ratio also shows a similar trend, with a greater contribution from *Sphagnum*, which prefers wetter conditions, at deeper depths, suggesting that restoration practices have been successful in recovering aquatic vegetation in both bogs. Conversely, at the BK-N and BK-ER sites, all samples show the highest P_aq_ values and ratios of C_23_/C_29_ and C_25_/C_29_ at the upper two layers, suggesting a slight shift in vegetation from more vascular-dominated plants to *Sphagnum* in the upper layer. This indicates successful restoration efforts in enhancing the presence of *Sphagnum* at these depths. The C_23_/C_25_ ratio indicates a higher contribution of *Sphagnum* species that prefer wetter conditions, generally dominating over those that prefer drier conditions.

According to Bingham et al.,^55^ species such as *S. medium/divinum*, *S. fuscum*, and *S. rubellum* are characterized by a maximum abundance of C_25_ and prefer drier conditions. However, *S. angustifolium* and *S. cuspidatum* have maxima at C_23_ and prefer wetter conditions. Therefore, in peat deposits, shifts in the C_23_/C_25_ ratio may reflect changes in the bog wetness over time and lower rations may support the establishment of hummocks in a functioning bog system. These findings highlight the importance of site-specific restoration strategies for effective ecosystem restoration.

### Influences of environmental factors on bacterial hopanoid biomarkers

Biohopanoids are pentacyclic triterpenoids produced by a wide range of bacteria and vascular plants^103^^,^^104^ and appear to have a regulatory and stiffening function similar to sterols in eukaryotes.^63^^,^^105^ These compounds can be divided into two groups: simple hopanoids with a C_30_ ring system (e.g., diploptene/diplopterol) and complex hopanoids such as bacteriohopanepolyols (BHPs), which have an additional polyfunctionalized side chain.^62^ Biohopanoids undergo a wide range of degradation processes, including functional groups loss, structural modification, and stereochemical, resulting in the formation of geohopanoids.^106^^,^^107^

In our study, biohopanoids such as hopanols, hopanes, and hopenes, were dominant in raised bog peat. The high abundance of biohopanoids 17β(H),21β(H)-21-hopane in peat cores in our study sites, especially in PU-N and PI-ER, could be related to the presence of aerobic methanotrophic bacteria. According to a study by Raghoebarsing et al.,^108^ methane is absorbed by submerged *Sphagnum* mosses in association with partially endophytic methanotrophic bacteria. This effectively recycles methane *in situ*, reducing methane emissions from *Sphagnum*-dominated peat bogs. Kip et al.^109^ also observed mitigation of methane emissions from *Sphagnum cuspidatum*-dominated ombrotrophic peat bogs, suggesting *Sphagnum* and its methane-oxidizing bacteria serve as an *in situ* emission filter. The presence of 17β(H),21β(H)-hopanol indicates the presence of aerobic methanotrophic bacteria particularly those symbiotic with *Sphagnum* mosses, including *S. cuspidatum*, which is present at our study sites.^110^

Interestingly, at the BK-N and BK-ER, the dominance of αβ-hopane over the distribution of ββ hopane seems to be strongly correlated within acidic (pH < 6), ombrotrophic, and *Sphagnum-*dominated peats.^62^ Furthermore, the presence of *Sphagnum* seems to stimulate the isomerization of the αβ-C_31_ hopane, leading to an increased rate of synthesis.^111^ This suggests that high acidity is an important factor in these conditions, but it may not fully explain the formation of αβ-hopanoids, particularly, in the absence of *Sphagnum* or in highly decomposed peat sites like AV-D and AV-ED. Furthermore, *n*-alkane data also supports the role of *Sphagnum*-dominated environments on organic matter composition. The higher concentrations of mid-chain *n*-alkanes (C_23_–C_25_) at wetter, *Sphagnum*-rich sites further confirm *Sphagnum* mosses as major contributors to the organic matter. These results highlight the complex interaction between *Sphagnum*-associated microbial communities and the organic matter dynamics in peatland ecosystem.

Furthermore, Huang et al.^112^ showed that in dry environments, hydrological conditions can increase geohopanoid isomerization. While hydrology and pH are essential aspects of peat ecosystems, heavy rainfall can cause dilution that decreases acidity, which may decrease αβ hopanoid formation, likely due to less favorable conditions for the bacteria involved in their formation.^62^^,^^113^ These findings underscore the importance of hydrology and pH in hopanoid formation and are essential for understanding peatland ecosystems. As Zhang et al.^83^ proposed, hopanoids derived from aerobic bacteria can serve as valuable biomarkers to reconstruct hydrological conditions in peatlands, which is a particularly important issue related to global climate change. However, further research is needed to fully understand how hydrological fluctuations influence hopanoid formation.

#### Archaeol

Archaeol serves as an important biomarker for many archaea, especially anaerobic methanogenic archaea, known to produce significant amounts of archaeol under anoxic conditions and typically found below the water surface.^114^^,^^115^ This biomarker has been used in the past to measure redox conditions in peatlands, particularly to investigate the effects of water table fluctuations on methane (CH_4_) production.^116^^,^^117^^,^^118^ According to Pancost et al.,^119^ archaeol is absent in the surface layers of peatlands, appearing between the maximum and minimum water table depths and extending further downwards.

At the AV-D and AV-ED sites, despite both bogs being currently dry, diploptene, and archeol are still detectable at both shallower and deeper depths. Only moderate levels of archaeol, considered below the detection limit by Pancost et al.^119^ were detected at 10 and 30 cm depths in the AV-ED site. This may be due to anaerobic conditions that can occur in small areas (microsites) of the temporarily saturated or wet soil, which could enable archaea to thrive at shallower depths.^117^ However, higher archaeol concentrations occur at deeper depths, as methanogen activity typically occurs at or below the water table rather than at the peat surface.^120^ The presence of diploptene (aerobic conditions) and archaeol (anaerobic conditions) at these sites highlights the complexity of redox conditions in these peat bogs and suggests that both aerobic and anaerobic processes remain active even in dry conditions.

At PU-N and PI-ER sites, the presence of diploptene and archaeol suggests the coexistence of both oxic and anoxic conditions. At PU-N, archaeol is present in low concentrations in the oxic layer, but its levels increase significantly at greater depth. At 80 cm depth, archaeol reaches approximately 60 μg/g and 159 μg/g in the PU-N and PI-ER sites, respectively, compared to a maximum of up to 40 μg/g observed in ombrotrophic British bogs.^119^

Conversely at the BK-N and BK-ER sites, archaeol concentrations are exceptionally high at deeper depths, specifically at the interface between the acrotelm and catotelm. Pancost et al.^119^ found that in four ombrotrophic European peatlands, archaeol was detected below the water table (catotelm), but was most concentrated at the interface between the oxic and anoxic layers. In ombrotrophic bogs, this is because methanogenesis is dependent on substrate availability, which is highest at the interface.^121^^,^^122^ Additionally, the BK-N site shows the presence of diploptene at depths of 30 and 80 cm, whereas it is completely absent at the BK-ER site. The highest concentration of mid-chain *n*-alkanes (C_23_–C_25_) at 80 cm depth in BK-N further supports the potential difference in microbial contribution and organic matter composition between sites. While diploptene is primarily produced by aerobic organisms, it can also be synthesized by anaerobic microorganisms. Further research is needed to explore the relationship between archaeol concentration and water table dynamics.

### Conclusion

In conclusion, the *n*-alkane analysis of peat bogs reflects the highest contribution from both vascular plants and *Sphagnum* mosses, as evidenced by the dominance of medium-to long-chain *n-*alkanes. This finding underscores the capability of these biomarkers to effectively reflect the current vegetation within peatlands and provides valuable insights in current conditions within these ecosystems. The use of *n*-alkane indices such as CPI, ACL, P_aq_, and vegetation ratios (C_23_/C_29_, C_25_/C_29_, and C_23_/C_25_) further illustrate shifts in vegetation types and moisture conditions, highlighting the sensitivity of peatland ecosystems to environmental changes, and the ability of biomarkers to track such shifts over time. Additionally, the analysis of steroids and pentacyclic triterpenoids has given us a clearer understanding of peatland ecology, revealing again detailed information about changes in vegetation and the conditions that facilitate preservation of peat organic matter. Moreover, microbial biomarkers, such as hopanoids and archaeol throughout the peat layers highlight their value in understanding a wide range of bacterial and archaeal activities and provide further insights into the redox conditions in peatlands, illustrating their potential for tracking microbial processes across different environmental conditions. Collectively, these biomarkers demonstrate their suitability as reliable indicators of current ecological conditions and provide a basis for future studies incorporating more extensive data on site histories on peatland restoration.

Our results indicate that restoration practices have successfully recovered the vegetation in sites like PI-ER and BK-ER. However, the lower biomarker concentrations including vegetation and microbial contribution observed in AV-D and AV-ED confirm that additional efforts are required to maintain and restore these sites closer to natural conditions. Overall, this study highlights the complex interactions between biological, chemical, and environmental factors that shape the biomarker profile in peatlands. It also highlights the importance of developing restoration strategies that recognize the complex ecological processes involved and the need for ongoing management to ensure the long-term stability and health of restored peatland ecosystems.

### Limitations of the study

This study provides valuable insights into the use of lipid biomarkers to assess peatland composition, decomposition and restoration success; however, it has several limitations. Only one peat core was collected per site, and biomarker analysis was conducted at three fixed depths (10, 30, and 80 cm), which limits both vertical and spatial resolution. As a result, finer-scale variability in biomarker distribution may not have been fully captured. Although water table levels were recorded, continuous environmental data, such as redox potential, temperature, or precipitation were not available. The lack of these data limits the ability to fully interpret the environmental context of the observed biomarker patterns. A broader and more detailed dataset on site histories, such as land use changes, drainage, and exact restoration measures is also lacking. This makes it difficult to fully connect biomarker signals with past ecological changes. Furthermore, the study focused on a limited set of lipid biomarkers; including a broader range of chemical indicators could provide a more complete understanding of peatland dynamics.

## Resource availability

### Lead contact

Further information and requests should be directed to the lead contact, Nasreen Jeelani (nasreen.jeelani@univie.ac.at).

### Materials availability

This study did not generate new unique reagents.

### Data and code availability

• **Data**: The published article includes all data generated or analyzed during this study. No additional datasets were generated.
• **Code**: This study did not generate any custom code.
• **Other**: No additional information is available.

## Acknowledgments

The project “ReVersal” was coordinated by K.-H.K. and S.G. Our special thanks go to Beatrix Bethke from the Geoecology Laboratory at Vienna University for her invaluable guidance on biomarker analysis. We are also grateful to Daniel Birgel for his assistance in the Hamburg laboratory. Additionally, we thank the Moorschutzverein Pürgschachen for granting us access to the Pürgschachen Moor site, a valuable part of the LTER network. Their contributions were essential to the success of this study.

This research was funded through the 2020–2021 Biodiversa and Water JPI joint call for research projects, under the BiodivRestore ERA-NET Cofund (GA N°101003777), with the 10.13039/100006826EU and the funding organizations 10.13039/501100002428FWF 10.13039/501100002428Austrian Science Fund (10.55776/15750), 10.13039/501100001659DFG (Germany), 10.13039/501100004281NCN (Poland), and the Ministry of LNV (The Netherlands). Open access funding was provided by 10.13039/501100003065University of Vienna.

## Author contributions

N.J.: Writing – review and editing, writing-original draft, visualization, investigation, formal analysis, methodology, conceptualization; K.F.: investigation, formal analysis, and methodology; C.L.T.: writing – review and editing; K.-H.K.: writing – review and editing; M.L.: writing – review and editing; M.G.: writing – review and editing; S.G.: writing – review and editing, supervision, resources, funding acquisition, conceptualization.

## Declaration of interests

The authors declare no competing interests.

## STAR★Methods

### Key resources table

**Table:** 

REAGENT or RESOURCE	SOURCE	IDENTIFIER
**Chemicals, Peptides, and Recombinant Proteins**		

Squalane	Sigma-Aldrich	CAS 111-01-3
1-Nonadecanol	Sigma-Aldrich	CAS 1454-84-8
Sodium sulphate (NaSO_4_)	Sigma-Aldrich	CAS-7757-82-6
BSTFA bis(trimethylsilyl)trifluoroacetamide)	Sigma-Aldrich	CAS 25561-30-2

**Other**		

GC-MS	Agilent Technologies	Model 7890A + 5975 MSD

**Biological Samples**		

Peat samples	1. Amtsvenn-Hündfelder Moor (Germany) 2. Pürgschachen Moor and Pichlmaier Moor (Austria) 3. Bagno Kusowo (Poland)	Field sampling

**Software and Algorithms**		

MassHunter	Agilent	Version 10.0
Excel	Microsoft	Microsoft 365 (2024 version)
NIST	Mass Spectral Library	20.0
SigmaPlot	Systat Software Inc.	13.0
CorelDraw	Graphics and design	2023 version

### Experimental model and study participant details

The study did not involve any animal or human clinical subject research. Peat cores were collected with a Russian peat corer from natural, extracted, degraded, and restored bogs across three countries; Amtsvenn, Germany; Bagno Kusowo, Poland; and Pichlmaier Moor, Pürgschachen Moor, Austria in Germany, Poland, and Austria. At each location, we chose two study sites and sampled peat from 10, 30 and 80 cm depth to analyze the biomarker signals. The depths were chosen to include distinct peat layers with differing redox conditions: the 10 cm depth corresponds to the acrotelm, which is primarily in oxic conditions; the 80 cm depth represents the catotelm, which remains permanently waterlogged and anoxic, even at the drained sites under study; and the 30 cm depth represents the zone between the acrotelm and catotelm, where water table fluctuations can lead to change between oxic and anoxic conditions. Samples were packed and transported in plastic bags, and later stored at −18°C.

### Method details

In preparation for lipid biomarker analysis, peat samples were freeze-dried and ground with mortar and pestle before extraction. Internal standards were added before extraction to correct for recovery efficiency (100 μL each): squalane (CAS-111-01-3), 0.81 μg/ml for the hydrocarbons, and 1-nonadecanol (CAS 1454-84-8), 1000 μg/mL for alcohols. The samples were saponified with 50 mL of potassium hydroxide (6% in methanol) for 2 h at 70°C in the oven, centrifuged multiple times, and then decanted into a separating funnel. We extracted about 2–3 g of dried peat by ultra-sonication (sonic bath) for 15 min using a 3:1 mixture of methanol and dichloromethane. After each step, we centrifuged the sample at 2500 rpm for 5 min, decanted off the clear extract, and poured the resulting solution into the separation funnel. This process was repeated three times until the solution became clear. The sample was shaken after being acidified to pH 1 with 10% hydrochloric acid and by adding about 40 mL of water. Two phases were clearly visible, the organic phase was cleaned by running through a filter with glass wool and sodium sulfate (NaSO4) (CAS-7757-82-6). All the extracts were combined and concentrated on a rotary evaporator under reduced pressure before transferring them to a small vial. The remaining solvent was evaporated under a nitrogen stream to dryness, and the solid sample residue was weighed. As the samples still contained asphaltenes, they were diluted with hexane and filtered again through a glass wool and NaSO4 filter several times. In this step, the maltenes were separated from the asphaltenes. The maltenes were further separated into the final fractions of hydrocarbons and alcohols fractions.

The maltene-fraction as mobile phase was applied on an aminopropyl column as stationary phase. The fractions were eluted with hexane for the *n*-alkanes and with dichloromethane and acetone (9:1) for the alcohol fraction. The alcohol fraction was derivatized with 100 μL of BSTFA (N,O-bis(trimethylsilyl)trifluoroacetamide) at 70°C for 1 h to improve volatility and detectability.

We used a GC-MS Agilent 7890A combined with 5975-MSD quadrupole mass spectrometer to identify and quantify n-alkanes. The GC was fitted with an HP-5MS Ultra inert capillary column (30 m length × 0.25 mm) with a film thickness of 0.25 μm. Helium with 99.9% purity was used as a carrier gas. The sample was injected in splitless mode setting the injector temperature at 310°C. The oven temperature was initially at 60°C and held for 1 min, then ramped to 150°C at a rate of 10°C/min. Finally, we ramped to 325°C at a rate of 4°C/min and kept the temperature constant for 35 min. The compounds were identified by comparing their retention times and mass spectra to those of reference compounds.

### Quantification and Statistical analysis

Biomarker concentrations were calculated to dry weight and expressed in micrograms per gram of dry weight (μg/g dry wt). Descriptive statistics were calculated using Microsoft Excel to summarize concentrations across sites and depths. Peak detection and quantification were carried out using Agilent ChemStation and MassHunter software. Compound identification was based on retention time and fragmentation patterns, using the National Institute of Standards and Technology (NIST) Mass Spectral Library and a lipid-specific library. The graphs were plotted and fitted using SigmaPlot and CorelDraw software.
